# Disinfection of Ebola Virus in Sterilized Municipal Wastewater

**DOI:** 10.1371/journal.pntd.0005299

**Published:** 2017-02-01

**Authors:** Kyle Bibby, Robert J. Fischer, Leonard W. Casson, Nathalia Aquino de Carvalho, Charles N. Haas, Vincent J. Munster

**Affiliations:** 1 Department of Civil and Environmental Engineering, University of Pittsburgh, Pittsburgh, Pennsylvania, United States of America; 2 Department of Computational and Systems Biology, University of Pittsburgh, Pittsburgh, Pennsylvania, United States of America; 3 Laboratory of Virology, Division of Intramural Research, National Institute of Allergy and Infectious Diseases, National Institutes of Health, Hamilton, Montana, United States of America; 4 Department of Civil, Architectural & Environmental Engineering, Drexel University, Philadelphia, Pennsylvania, United States of America; University of California Berkeley, UNITED STATES

## Abstract

Concerns have been raised regarding handling of Ebola virus contaminated wastewater, as well as the adequacy of proposed disinfection approaches. In the current study, we investigate the inactivation of Ebola virus in sterilized domestic wastewater utilizing sodium hypochlorite addition and pH adjustment. No viral inactivation was observed in the one-hour tests without sodium hypochlorite addition or pH adjustment. No virus was recovered after 20 seconds (i.e. 4.2 log_10_ unit inactivation to detection limit) following the addition of 5 and 10 mg L^-1^ sodium hypochlorite, which resulted in immediate free chlorine residuals of 0.52 and 1.11 mg L^-1^, respectively. The addition of 1 mg L^-1^ sodium hypochlorite resulted in an immediate free chlorine residual of 0.16 mg L^-1^, which inactivated 3.5 log_10_ units of Ebola virus in 20 seconds. Further inactivation was not evident due to the rapid consumption of the chlorine residual. Elevating the pH to 11.2 was found to significantly increase viral decay over ambient conditions. These results indicate the high susceptibility of the enveloped Ebola virus to disinfection in the presence of free chlorine in municipal wastewater; however, we caution that extension to more complex matrices (e.g. bodily fluids) will require additional verification.

## Author Summary

Ebola virus infected individuals may generate up to nine liters of potentially infectious liquid waste per day. Previous recommendations were to directly dispose of this waste into a sanitary sewer or latrine; however, release of infectious virus raised the concern of environmental transmission through unintentional contact with contaminated wastewater. One possibility to reduce or eliminate the release of infectious virus is disinfection of Ebola virus contaminated liquid waste. A hurdle to making recommendations for liquid waste disinfection is the lack of data on disinfection efficacy. Here we demonstrate that Ebola virus in municipal wastewater is highly sensitive to disinfection in the presence of free chlorine. In addition, elevating the pH to 11.2 significantly increased the rate of decay over neutral pH conditions. These results provide a basis to develop recommendations for the disinfection of Ebola virus contaminated wastewater.

## Introduction

Ebola virus infected individuals shed the virus in bodily fluids [1–3] and may produce up to nine liters of bodily waste per day, in addition to wash waters [4]. Subsequently, concerns were raised during the 2014/15 Ebola virus epidemic regarding the appropriate handling of Ebola virus contaminated wastewater to minimize potential secondary exposure to the virus [5]. Ebola virus is an enveloped filovirus that is primarily spread via direct contact with infected individuals [6]. Secondary transmission via environmental routes (i.e. fomites) has previously been recognized [7], but the available evidence on environmental transmission is controversial [8]. Previously reported concentrations of Ebola virus in bodily fluids (sweat, urine, and stool) has been in the range of 2.8–7.2 log_10_ viral RNA copies mL^-1^ [9–11], and 5 log_10_TCID_50_ mL^-1^ in the blood of infected macaques [12]. The conversion of RNA copies to viable virus is unknown. The median infectious dose for Ebola virus is low, in the range of nine plaque forming units, depending on the route of infection [13].

The World Health Organization initially recommended that liquid waste from Ebola patients be directly disposed into the sanitary sewers or latrines without disinfection [5]. The recommendation for direct disposal of Ebola virus contaminated liquid waste was made due to the expected rapid inactivation and dilution of Ebola virus in wastewater, as well as a lack of evidence for Ebola virus transmission via water. Subsequently, questions were raised regarding Ebola virus persistence in wastewater and appropriate approaches for disinfection. Research has since identified the T_90_ (time for 90% inactivation) of Ebola virus in sterilized wastewater to be 2.1 days [14], which is consistent with estimated persistence using viral surrogates [15]. Additionally, waste, including wastewater, has since been highlighted as a possible transmission risk—especially waste contaminated with infected blood [16]. Previous evaluations have demonstrated that Ebola virus is highly stable in blood [17]. In response to the uncertainty regarding appropriate wastewater disinfection approaches and the resulting risk of secondary exposure or transmission, Ebola Treatment Units in the United States chose *ad hoc* liquid waste disinfection approaches prior to disposal [4]. The World Health Organization ultimately revised recommendations to suggest holding liquid waste in latrines for a week to allow viral decay and inactivation [18].

Currently, the disinfection kinetics of Ebola virus in liquid is unknown. In a previous evaluation of Ebola virus disinfection on surfaces, sodium hypochlorite at 0.01% and 0.1% was found to be ineffective but 0.5% and 1% sodium hypochlorite removed viable virus in five minutes [19]. Additionally, filoviruses have been previously recognized to be highly susceptible to inactivation by UV exposure [20, 21]. The pH stability of Ebola virus in wastewater is unknown.

The overarching study goal was to determine the disinfection of Ebola virus in municipal wastewater, of direct relevance to wastewater management in an outbreak scenario. Our scope was limited to municipal wastewater and did not consider the disinfection of Ebola virus in concentrated human waste (e.g. feces, vomit, or blood). It should be noted that disinfection under high organic load (e.g. feces, vomit, or blood), which is not the focus of the current manuscript, would require hyper-chlorination, which has been suggested to inconsistently achieve adequate disinfection and would require additional experimental verification [22]. In the current study we evaluated the disinfection of Ebola virus in sterilized domestic wastewater by chlorine addition and pH adjustment. Study limitations as well as implications for wastewater handling in outbreak response are discussed.

## Methods

Wastewater samples were collected from a municipal wastewater treatment plant as described previously [14] and shipped overnight on ice to Rocky Mountain Laboratories. Upon receipt, samples were sterilized with five mega-rads of gamma irradiation and a subset of gamma-irradiated sample was sent back to the University of Pittsburgh for characterization and chlorine demand analysis. Wastewater characteristics are summarized in Table 1. Sterilization was performed to block microbial growth during cell culture, which would make virological analyses impossible. Stock virus (Ebola virus Guinea Makona-WPGC07, 10^7.3^ TCID_50_ mL^-1^) [23] was diluted in wastewater to achieve an approximate starting viral titer of 10^5^ TCID_50_ mL^-1^ for both Ebola virus disinfection experiments and pH inactivation experiments. All experiments were completed in triplicate at 20°C. Ebola virus titration and cultivation were performed as previously described [14]. The limit of detection for all replicates was 0.75 log TCID_50_ mL^-1^.

**Table 1:** Composition of gamma irradiated wastewater. Values in brackets indicate 95% confidence interval.

Constituent	Wastewater
pH	6.9
Chemical Oxygen Demand (mg/L)	54.7(± 3.5)
Ammonia (mg/L)	32.5 (± 2)
Total Organic Carbon (mg/L)	31.6 (± 4.3)
Total Suspended Solids (mg/L)	129 (± 9)

For disinfection experiments, sodium hypochlorite (Acros Organics) was added to two milliliter vials of the wastewater/virus suspension at initial doses of 0, 1, 5, and 10 mgL^-1^. Samples were then taken at the indicated time points and chlorine demand immediately quenched by the addition of sodium thiosulfate. The ‘time zero’ sampling point was taken approximately 20 seconds following the addition of chlorine to enable sample mixing.

Three pH values were evaluated for pH inactivation experiments: 6.9 (intrinsic), 4.3, and 11.2. pH values were found to be stable for the time period evaluated. The tested pH values were chosen to be below the previously recognized Ebola virus glycoprotein stability down to pH = 4.8 [24] and to be within the tested values for sterilization of wastewater in an outbreak setting via elevated pH [22]. The virus was then directly added to the pH-adjusted wastewater, mixed via pipetting, and sampled. The ‘time zero’ sampling point was taken approximately 20 seconds following the addition of virus to enable sample mixing.

Chlorine residuals in both the untreated and the gamma-irradiated wastewater were experimentally determined outside of the Biosafety Level 4 facility using a Hach Free Chlorine test kit (method 10069) in triplicate. Chlorine residual was experimentally found to be dose dependent (S1 Fig). To determine the immediate chlorine demand (and residual), chlorine residual was plotted versus time for each initial chlorine dose. A linear fit was then applied to each the residual versus time plot for each dose, and the y-intercept (i.e. modeled initial chlorine residual) of the linear fit was determined (S2–S4 Figs). Chlorine residuals of zero were excluded from this fit. Chlorine decay was then modeled as previously described eq (1) [25];

$$\text{C}={\text{C}}_{0}e^{-\text{kt}}$$ (1)

C_0_ was the modeled initial chlorine residual. The concentration-time exposure was then calculated for each sampling time point by integrating the area under the modeled chlorine residual curve at each time point.

Statistical analyses and graphing were completed with Prism 7.0a and Microsoft Excel 2011.

## Results and Discussion

**Fig 1:**
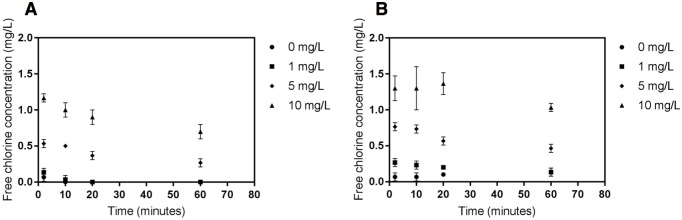
Free chlorine concentration in sterilized (gamma-irradiated) wastewater (A) and wastewater (B) over four time points (2, 10, 20 and 60 minutes) for initial doses of 0, 1, 5, and 10 mgL^-1^ sodium hypochlorite. Error bars represent the standard deviation calculated from three replicates.

To examine disinfection kinetics, chlorine residual was modeled based upon laboratory measures of free chlorine in the gamma-irradiated wastewater without viral addition. Measured free chlorine concentrations for both the unsterilized and gamma-irradiated wastewater are shown in Fig 1. The effect of sterilization on chlorine demand was statistically significant for all concentrations (*p* < 0.05); however, the chlorine decay was more rapid in irradiated wastewater compared to the wastewater without irradiation, suggesting that testing in the irradiated wastewater would demonstrate less rapid viral inactivation than would be observed in the non-irradiated wastewater at the same applied dose. Chlorine residual was found to be dose-dependent (S1 Fig), and modeling of immediate chlorine demand in the sterilized wastewater determined that 0, 1, 5, and 10 mg L^-1^ doses resulted in initial free chlorine residuals of 0, 0.16, 0.52, and 1.11 mg L^-1^, respectively. As Ebola virus inactivation was expected to be rapid, the chlorine dosing conditions were selected to provide a range of representative free chlorine concentrations while capturing inactivation kinetics of Ebola virus. The chlorine residual measurements and model results are shown in S5 Fig. Concentration-time (Ct) values were then determined by integrating the area under the chlorine residual curve at each sampling time point.

**Fig 2:**
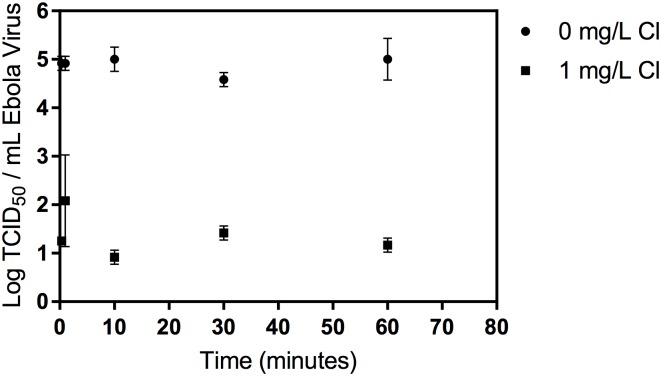
Disinfection of Ebola virus at 0 and 1 mg/L added chlorine. No virus was recovered from the 5 and 10 mg/L chlorine conditions at any time point. The limit of detection was 0.75 log TCID_50_/mL. Error bars represent one standard deviation.

No decay of the virus was observed for the 0 mg L^-1^ condition during the one-hour test. No virus was recovered at any time point for the 5 and 10 mg L^-1^ chlorine from the 10^5^ TCID_50_ mL^-1^ starting virus concentration (maximum observable reduction 4.18 log_10_ TCID_50_ mL^-1^). Data for the 0 and 1 mg L^-1^ conditions from the 10^5^ TCID_50_ mL^-1^ virus concentration is shown in Fig 2 and S1 Table. Following an initially rapid viral inactivation (approximately 3.5 log_10_ TCID_50_ mL^-1^ in 20 seconds), no further viral removal was observed in the 1 mg/L condition, likely due to the low concentration of free chlorine (S5 Fig).

**Fig 3:**
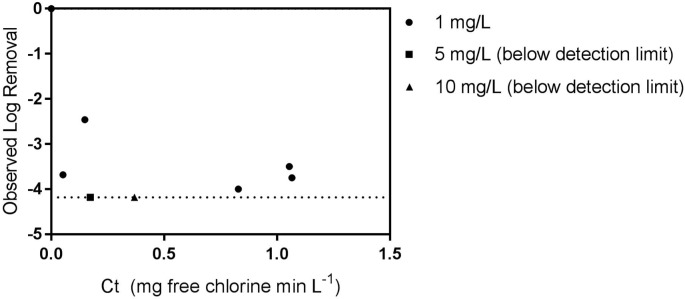
Log viral removal versus estimated Ct. Viral removal for 5 and 10 mg L^-1^ chlorine conditions shown at detection limit for demonstration purposes. Dashed line represents limit of detection. The limit of detection was 0.75 log TCID_50_ mL^-1^, resulting in a maximum observable log_10_ TCID_50_ removal of 4.18 TCID_50_ mL^-1^.

Observed viral inactivation versus calculated Ct is shown in Fig 3. For demonstration, values are plotted at the limit of viral detection for the 5 mg L^-1^ and 10 mg L^-1^ dosing conditions, although no virus was recovered. The most persistent observed inactivation to achieve four log_10_ units of Ebola virus removal was equivalent to 1.1 mg-min L^-1^. The current US EPA recommendation to achieve four logs of virus removal in drinking water at the tested conditions is 3 mg-min L^-1^ of free chlorine [26]. These results demonstrate that requirements for Ebola virus disinfection would be expected to be at three-fold below current standards for virus disinfection in water; however, the chlorine demand of the wastewater being disinfected must first be exceeded for this recommendation to be valid.

It was noted that Ebola virus from later time points in the 1 mg L^-1^ experiment appear to be more resistant based upon calculated Ct values than would be estimated by initial Ebola virus disinfection time points or observed removal at the 5 and 10 mg L^-1^ doses. This observed persistence effect may be due to multiple factors, such as particle association or aggregation of the virus providing some protection from disinfection [27, 28] or incomplete mixing of the added chlorine and rapid consumption of the available free chlorine residual. Alternatively, there may potentially be a ‘persistent’ Ebola virus population that may be more disinfectant resistant. Finally, there may have been a more rapid decay of the chlorine residual than modeled (perhaps due to the chlorine demand of the viral suspension).

**Fig 4:**
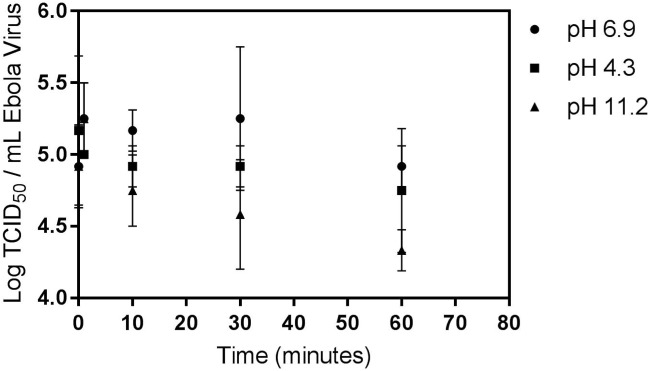
Ebola virus persistence in wastewater at neutral (6.9), acidic (4.3), and basic (11.2) pH. The limit of detection was 0.75 log TCID_50_ mL^-1^. Error bars represent one standard deviation.

We also investigated Ebola virus inactivation via pH adjustment. Results are shown in Fig 4 and S2 Table. No viral inactivation was observed within the test period at the ambient wastewater pH of 6.9. No statistically significant inactivation was observed at pH 4.3. At pH 11.2 and with a 10^5^ TCID_50_ mL^-1^ starting virus concentration, the 95% confidence interval for one log inactivation was found to be 52 to 193 minutes.

### Limitations

The current study has multiple limitations. The wastewater was required to be disinfected by gamma irradiation to avoid bacterial contamination and toxicity of the cell culture line. This irradiation resulted in an increased chlorine demand by the wastewater and may have altered other wastewater chemistry. Additionally, chlorine residual modeling was performed based upon laboratory tests using solely the gamma-irradiated wastewater. The virus to be disinfected was suspended in cell culture media—while the virus suspension comprised less than 1% of the test matrix, this has the potential to alter the chlorine demand of the test. We note that in this case, the observed viral persistence would be conservative, i.e. the actual Ct would in fact be less than the modeled Ct for the evaluated conditions and viral inactivation would be more rapid than reported. Due to the dilute nature of the wastewater evaluated, the role of higher organic loading and particle association in protecting virus from disinfection remains unresolved, although recent studies using Ebola virus surrogates have suggested that the majority (~90%) of viral particles remain not particle associated [29, 30].

### Implications

Despite the end of the most recent Ebola virus epidemic, concerns remain regarding the potential transmission of emerging enveloped viruses via water [31], highlighting the value of continued investigation into enveloped virus persistence and disinfection. These results demonstrate the high susceptibility of Ebola virus to disinfection in the presence of free chlorine. The most conservative estimate for Ebola virus disinfection was less than current recommendations for waterborne virus inactivation, suggesting that existing disinfection approaches are adequate to achieve Ebola virus reductions in wastewater. In addition, elevated pH would provide significantly improved viral inactivation over ambient decay. These results highlight the value of considering wastewater disinfection in response to infectious disease outbreaks to minimize the risk of secondary transmission, as well as to address public concern.

## Supporting Information

S1 Table Raw data for each replicate for hypochlorite inactivation experiment at a target starting concentration of 5 log TCID_50_ mL^-1^. All values log TCID_50_ mL^-1^. Limit of detection for each replicate was 0.75 log TCID_50_ mL^-1^.
(DOCX)

S2 Table Raw data for each replicate at a target starting concentration of 10^5^ TCID_50_ mL^-1^. All values log TCID_50_ mL^-1^. Limit of detection for each replicate was 0.75 log_10_ TCID_50_ mL^-1^.
(DOCX)

S1 Fig Chlorine residual versus applied dose at measured time points. (DOCX)

S2 Fig Chlorine residual versus time for 1 mgL^-1^ dose condition. No residual detected at later time points.
(DOCX)

S3 Fig Chlorine residual versus time for 5 mgL^-1^ dose condition. (DOCX)

S4 Fig Chlorine residual versus time for 10 mgL^-1^ dose condition. (DOCX)

S5 Fig Measured and modeled chlorine residuals versus time for added chlorine doses. (DOCX)
