# Supplementary material for: Disinfection of Ebola Virus in Sterilized Municipal Wastewater
Source: PLoS Negl Trop Dis. 2017 Feb 1;11(2):e0005299. doi: 10.1371/journal.pntd.0005299 (PMC5287448; doi:10.1371/journal.pntd.0005299)
Supplement: S1 Table — All values log TCID50 mL-1. Limit of detection for each replicate was 0.75 log TCID50 mL-1. (DOCX) [file pntd.0005299.s001.docx]

| **S1 Table.** Raw data for each replicate for hypochlorite inactivation experiment at a target starting concentration of 5 log TCID_50_ mL^-1^. All values log TCID_50_ mL^-1^. Limit of detection for each replicate was 0.75 log TCID_50_ mL^-1^. | | | | | | |
| --- | --- | --- | --- | --- | --- | --- |
| **Minutes** | **0 mgL^-1^ Cl** | | | **1 mgL^-1^ Cl** | | |
| 0.33 | 4.75 | 5.00 | 5.00 | 1.25 | 1.25 | 1.25 |
| 1 | 4.75 | 5.00 | 5.00 | 1.00 | 2.50 | 2.75 |
| 10 | 5.25 | 4.75 | 5.00 | 0.75 | 1.00 | 1.00 |
| 30 | 4.50 | 4.75 | 4.50 | 1.25 | 1.50 | 1.50 |
| 60 | 5.25 | 5.25 | 4.50 | 1.25 | 1.00 | 1.25 |
