# Supplementary material for: Disinfection of Ebola Virus in Sterilized Municipal Wastewater
Source: PLoS Negl Trop Dis. 2017 Feb 1;11(2):e0005299. doi: 10.1371/journal.pntd.0005299 (PMC5287448; doi:10.1371/journal.pntd.0005299)
Supplement: S2 Table — All values log TCID50 mL-1. Limit of detection for each replicate was 0.75 log10 TCID50 mL-1. (DOCX) [file pntd.0005299.s002.docx]

| **S2 Table.** Raw data for each replicate at a target starting concentration of 10^5^ TCID_50_ mL^-1^. All values log TCID_50_ mL^-1^. Limit of detection for each replicate was 0.75 log_10_ TCID_50_ mL^-1^. | | | | | | | | | |
| --- | --- | --- | --- | --- | --- | --- | --- | --- | --- |
| **Minutes** | **pH 6.9** | | | **pH 4.3** | | | **pH 11.2** | | |
| 0 | 4.75 | 4.75 | 5.25 | 4.75 | 5.00 | 5.75 | 4.75 | 5.25 | 4.75 |
| 1 | 5.25 | 5.50 | 5.00 | 5.00 | 5.00 | 5.00 | 5.50 | 5.00 | 5.25 |
| 10 | 5.25 | 5.00 | 5.25 | 5.00 | 4.75 | 5.00 | 4.50 | 5.00 | 4.75 |
| 30 | 5.75 | 5.25 | 4.75 | 5.00 | 5.00 | 4.75 | 4.25 | 5.00 | 4.50 |
| 60 | 5.00 | 5.00 | 4.75 | 5.00 | 4.25 | 5.00 | 4.50 | 4.25 | 4.25 |
