# Supplementary material for: Disinfection of Ebola Virus in Sterilized Municipal Wastewater
Source: PLoS Negl Trop Dis. 2017 Feb 1;11(2):e0005299. doi: 10.1371/journal.pntd.0005299 (PMC5287448; doi:10.1371/journal.pntd.0005299)
Supplement: S3 Fig — (DOCX) [file pntd.0005299.s005.docx]

**S3 Figure.** Chlorine residual versus time for 5 mgL^-1^ dose condition.
